# Postural sway in multiple sclerosis patients: interaction of vision, surface, and fatigue effects

**DOI:** 10.3389/fnhum.2025.1624969

**Published:** 2025-10-21

**Authors:** Žiga Kozinc, Eva Žura, Gregor Brecl Jakob

**Affiliations:** ^1^Faculty of Health Sciences, University of Primorska, Izola, Slovenia; ^2^Department of Neurology, University Medical Centre Ljubljana, Ljubljana, Slovenia; ^3^Faculty of Medicine, University of Ljubljana, Ljubljana, Slovenia

**Keywords:** balance control, sensory integration, proprioception, motor impairments, fall prevention, neurological disorders, physical fatigue

## Abstract

**Introduction:**

Postural control impairments are common in patients with multiple sclerosis (MS), resulting in postural instability and increased fall risk. Sensory inputs are crucial to maintain balance adequately. Additionally, fatigue is one of the common and most disabling symptoms of MS, possibly contributing to postural deficits. Previous studies have examined the effects of fatigue and altered sensory conditions on postural control in patients with MS. The present study aimed to extend this knowledge by jointly assessing these factors within the same experimental framework, providing additional insight into how fatigue modulates sensory contributions to balance.

**Methods:**

A total of 21 patients with MS (age = 41.1 ± 10.1 years; EDSS = 1.9 ± 1.0; disease duration = 6.8 ± 4.9 years) completed balance assessments on firm and compliant surfaces with both eyes open and eyes closed, before and after a 6-min walk test used to induce fatigue. Postural sway was quantified using sway velocity and root mean square (RMS).

**Results:**

There was a significant effect of surface on sway velocity (*p* < 0.001, η^2^ = 0.60), with a greater sway on the compliant surface compared to the firm surface. Fatigue significantly increased sway RMS (*p* = 0.023, η^2^ = 0.23) but did not affect sway velocity (*p* > 0.05). The absence of visual input (eyes closed) also significantly increased sway RMS (*p* = 0.001, η^2^ = 0.46). There was a significant interaction between surface and vision for sway RMS (*p* < 0.001, η^2^ = 0.54), with a larger effect of surface instability in the eyes-closed condition.

**Discussion:**

Patients with MS face increased challenges in maintaining postural control under conditions of fatigue, surface instability, and lack of visual input. Sway RMS may be more sensitive to these effects than sway velocity.

## 1 Introduction

Postural control is fundamental to human motor function, enabling individuals to maintain balance and respond to external disturbances (Ivanenko and Gurfinkel, 2018). Multiple factors, including fatigue, unstable surface conditions, and the unavailability of sensory inputs, can disrupt postural stability (Gebel et al., 2022; Šarabon et al., 2022; Sullivan et al., 2009). This is a particularly important area of investigation in patients with neurological conditions, including multiple sclerosis (MS) (Comber et al., 2018; Fritz et al., 2022). MS is characterized by demyelination of the central nervous system, which leads to the development of motor, cerebellar, sensory, and cognitive dysfunctions that adversely affect balance (Brecl Jakob et al., 2019; Comber et al., 2018; Prosperini and Castelli, 2018). Postural sway is typically increased in patients with MS compared to healthy individuals and is among the factors contributing to increased fall risk observed in this population (Cameron and Lord, 2010; Sun et al., 2019).

Fatigue is one of the most debilitating symptoms of MS (Oliva Ramirez et al., 2021). It can substantially limit participation in employment, social activities, and leisure pursuits, thereby reducing the quality of life (Mollaoğlu and Üstün, 2009; Oliva Ramirez et al., 2021), although it is not necessarily correlated with overall disease severity (Giovannoni, 2006). Muscle fatigue decreases the ability to maintain balance, resulting in greater postural sway, even in healthy populations (Jo et al., 2022). In patients with MS, the 6-min walk test (6MWT) is sufficient to impair both static and dynamic balance (Arpan et al., 2020; Jackson and Bigelow, 2013). Fatigue also affects cognitive processing, further exacerbating the difficulties in maintaining postural control (McLoughlin et al., 2015). Postural control requires the continuous integration of sensory inputs and motor outputs (Peterka, 2002), and when cognitive resources are limited, the ability to allocate attention to balance tasks is compromised. This dual burden can exacerbate instability in patients with MS (McLoughlin et al., 2015; Chamard Witkowski et al., 2019). Thus, it is not surprising that the effects of fatigue on postural sway are exacerbated in MS (Jallouli et al., 2022; Van Emmerik et al., 2010; Sedaghati et al., 2023). While the effects of fatigue on postural sway are well established, it is less clear how these changes manifest in patients with MS under different measurement conditions. In particular, the availability of visual information and the characteristics of the support surface may influence the extent to which fatigue affects balance.

Previous research suggests that individuals with MS tend to rely excessively on visual feedback to compensate for proprioceptive deficits (McLoughlin et al., 2015; Poh et al., 2017). Given the potential influence of fatigue on proprioception (Lattanzio et al., 1997; Paillard, 2012), the effects of fatigue on postural sway may be more pronounced in conditions without vision. Indeed, studies consistently report increased postural sway in the eyes-closed condition in patients with MS compared to healthy controls, whereas findings for the eyes-open condition are more variable (Comber et al., 2018). Furthermore, surface stability may also contribute to fatigue-related impairments in postural control. While studies consistently demonstrate reduced postural control on compliant/unstable surfaces compared to firm/stable surfaces in healthy young and older populations (Barbado Murillo et al., 2012; Hsiao et al., 2020; Lord and Menz, 2000), this aspect remains less explored in patients with MS. Interestingly, a recent study reported larger postural sway in patients with MS compared to controls over a spectrum of conditions, but the increase with the addition of unstable surface was similar in each group (Odom et al., 2021). One study found that, following a sit-to-stand task combined with a 6-min walking test, postural sway in people with MS increased more in the eyes-closed condition compared to the eyes-open condition (Jallouli et al., 2022). On the other hand, another study found no changes in postural sway in quiet stance when comparing patients with MS after a strength-exercise-based fatiguing protocol (Van Emmerik et al., 2010).

Understanding how fatigue, surface conditions, and visual input interact to influence postural sway is needed to better understand the impairments in postural control in patients with MS. While previous studies have examined the influence of fatigue on postural control in patients with MS (Sedaghati et al., 2023) and its interaction with individual sensory manipulations, little is known about how fatigue modulates postural stability under multiple, simultaneous sensory interactions. The aim of this study was to investigate the combined effects of fatigue and varying sensory conditions (surface instability and visual input) on postural control in people with MS. We hypothesized that fatigue would impair postural control in patients with MS, with stronger effects under unstable and no-vision conditions, including when these sensory challenges were combined. We did not pre-specify differential hypotheses for sway RMS and sway velocity, as prior findings on their relative sensitivity have been mixed (Browne and O’Hare, 2001; Van Emmerik et al., 2010; Raymakers et al., 2005).

## 2 Methods

### 2.1 Participants

The participants included patients with MS, managed and treated in the Neurology Clinic, University Medical Centre Ljubljana. The inclusion criteria were patients diagnosed MS by a neurologist at the University Medical Centre Ljubljana, those with an Expanded Disability Status Scale (EDSS) score between 1 and 4, those with self-reported symptoms of fatigue (confirmed in consultation with the treating neurologist), and those with the ability to walk at least 6 min without rest. The exclusion criteria were those aged below 18 and above 65; those relapsed in the last 3 months or who underwent therapy with corticosteroids in the last 3 months those with secondary diseases and conditions, i.e., fresh injuries, disease relapses, acute internal conditions; those with clinically significant spasticity or muscle weakness, as judged by the neurologist, and those using ambulatory assistive devices. Participants with acute or clinically significant visual impairments that could affect balance assessments were also excluded, whereas those with minor or corrected impairments (e.g., use of glasses or contact lenses) were included. All participants were informed about the purpose, course, and potential risks of this study and signed written informed consent before enrolment. The study was approved by the National Medical Ethics Committee of the Republic of Slovenia (0120-236/2023/4).

### 2.2 Study design and procedures

This was a single-visit study, with assessments of postural sway performed before and after a protocol designed to induce fatigue. Specifically, the protocol involved the 6MWT, a validated and highly reliable measure of functional endurance in MS, which is widely used in both clinical and research contexts and has been shown to reliably provoke motor fatigability (Leone et al., 2016; Chen et al., 2021), which was the focus of this study. At the beginning of the testing session (before the fatigue protocol or any measurements), information about age, type of MS, Expanded Disability Status Scale (EDSS), height, and weight were collected. All assessments were carried out at the Neurology Clinic under the supervision of a licensed physical therapist and a board-certified neurologist, both with clinical experience in the management of MS. Static postural control was first assessed at baseline. After baseline testing, 6MWT was performed with the intention of inducing fatigue. The participants were instructed to walk as fast as possible, but in a safe manner. The rating of perceived exertion (RPE) was assessed immediately after completion of the 6-min walk test using the modified Borg Scale. After that, the postural control testing was repeated in the same way as it was before the walking test.

### 2.3 Postural sway measurement

Postural sway was measured using the Mobility Lab system (APDM, Inc., Portland, USA). Participants were wearing APDM Opal Sensors (APDM, Inc., Portland, USA) during the whole trial. These are small, lightweight inertial measurement units (dimensions 48.5 × 36.5 × 13.5 mm; weight ~22 g) equipped with triaxial accelerometers, gyroscopes, and magnetometers. Sensors were secured with elastic straps at the following anatomical sites: dorsum of the left and right wrists, aligned with the ulnar styloid process; dorsum of the left and right feet over the second metatarsal region (instep); anterior sternum at the manubrium level; and lumbar spine centred over the spinous process of L5. These sensors provide precise motion tracking in multiple planes of movement. Sensors were attached to predefined anatomical sites to assess postural control. Static postural control in parallel stance was assessed in four conditions (eyes opened-firm surface, eyes closed-firm surface, eyes opened-compliant surface, and eyes closed-compliant surface). Parallel stance was standardized as feet hip-width apart and arms relaxed alongside the body. Participants stood barefoot either on the laboratory floor (linoleum surface) or on a compliant foam pad (Airex Balance Pad, 50 × 41 × 6 cm). Each trial lasted 30 s, with participants asked to stand as still as possible. All trials were separated by seated rest periods of at least 1 min to minimize carryover fatigue. The order of conditions was randomly determined for each participant but was kept the same for each participant throughout the repeated testing. For eyes open conditions, the participants were asked to look into the marked point on the wall 3 m away, which was drawn in line with their eyes.

### 2.4 Outcome variables

To minimize the risk of false positive results and Type **I** error, we focused on only two outcome variables. Thus, the outcome measures considered were sway velocity and root mean square (RMS) sway. We chose sway RMS and sway velocity, which are commonly used parameters in MS research and have been shown to be sensitive indicators of balance impairments in this population (Browne and O’Hare, 2001; Van Emmerik et al., 2010; Raymakers et al., 2005). Data were wirelessly transmitted to the Mobility Lab software, which automatically processed the data. Sway velocity is calculated as the total displacement of the CoP (center of pressure) divided by the duration of the trial, while RMS sway was calculated as the square root of the mean of the squared deviations of the CoP from its mean position over the trial. Modified Fatigue Impact Scale (MFIS) (Larson, 2013) and Activities-specific Balance Confidence Scale (ABC) (Powell and Myers, 1995; Nilsagård et al., 2012) were used to assess fatigue-related impacts on daily activities and self-reported confidence in maintaining balance during various tasks, respectively. These tools provided a comprehensive evaluation of the participants’ perceived limitations and their confidence in performing functional movements without falling. These scales were not included in the main analysis but were employed to provide descriptive statistics of the patients, offering a clearer overview of the characteristics of our sample.

### 2.5 Statistical analysis

The data are presented as means ± standard deviations. The normality of the data distributions for all variables was verified with the Shapiro–Wilk test and visual inspection of histograms and Q-Q plots. A general linear model for repeated measures with fatigue (pre and post), vision (EO and EC), and surface (firm and compliant) was carried out to assess the main effects and interactions. To limit the risk of Type I error, only two primary sway outcomes (sway velocity and sway RMS) were analyzed. Accordingly, no further corrections for multiple comparisons (e.g., Bonferroni) were applied. Instead, effect sizes (partial η^2^) were reported alongside *p*-values to provide information about the magnitude and practical relevance of observed effects. Partial eta squared (ηp^2^) was calculated as the measure of effect size and was interpreted as small (~0.01), moderate (~0.06), or large (~0.14) (Bakeman, 2005). In case of statistically significant interaction effects, further *post-hoc* testing was done with unifactorial analyses of variance and paired-sample t-tests. In addition, estimated marginal means (EMM) with 95% confidence intervals (95% CI) were calculated. The threshold for statistical significance was set at *α* of <0.05, and all analyses were carried out in SPSS statistical software (version 25.0, IBM, USA).

## 3 Results

### 3.1 Participants characteristics

Twenty-one patients with MS, with a mean age of 41.1 (±10.1) years, a mean disease duration of 6.8 (± 4.9) years, and an EDSS of 1.9 (±1.0), were included in the study. The descriptive statistics of demographic data and 6MWT results are included in Table 1. The RPE scores at 6 min of 6MWT (10.14 ± 3.26, range = 6–17) indicated an induction of low to moderate fatigue. All 21 participants completed the study protocol, and no data were excluded as outliers. Consequently, all analyses were performed on the full sample.

**Table 1:** Descriptive statistics.

Variable	Mean	SD	Minimum	Maximum
Age (years)	41.1	10.1	23	64
Body height (cm)	168.8	8.4	152	190
Body mass (kg)	71.8	11.5	58	95
EDSS	1.92	1.00	0	4
Disease duration (years)	6.89	4.94	1	19
6MWT distance (m)	516.6	81.9	312	683
6MWT RPE at 3 min	8.71	2.95	4	15
6MWT RPE at 6 min	10.14	3.26	6	17
MFIS	35.52	21.47	5	72
ABC (%)	80.88	16.95	27.5	99.4

### 3.2 Interaction of vision, surface, and fatigue effects

**Figure 1:**
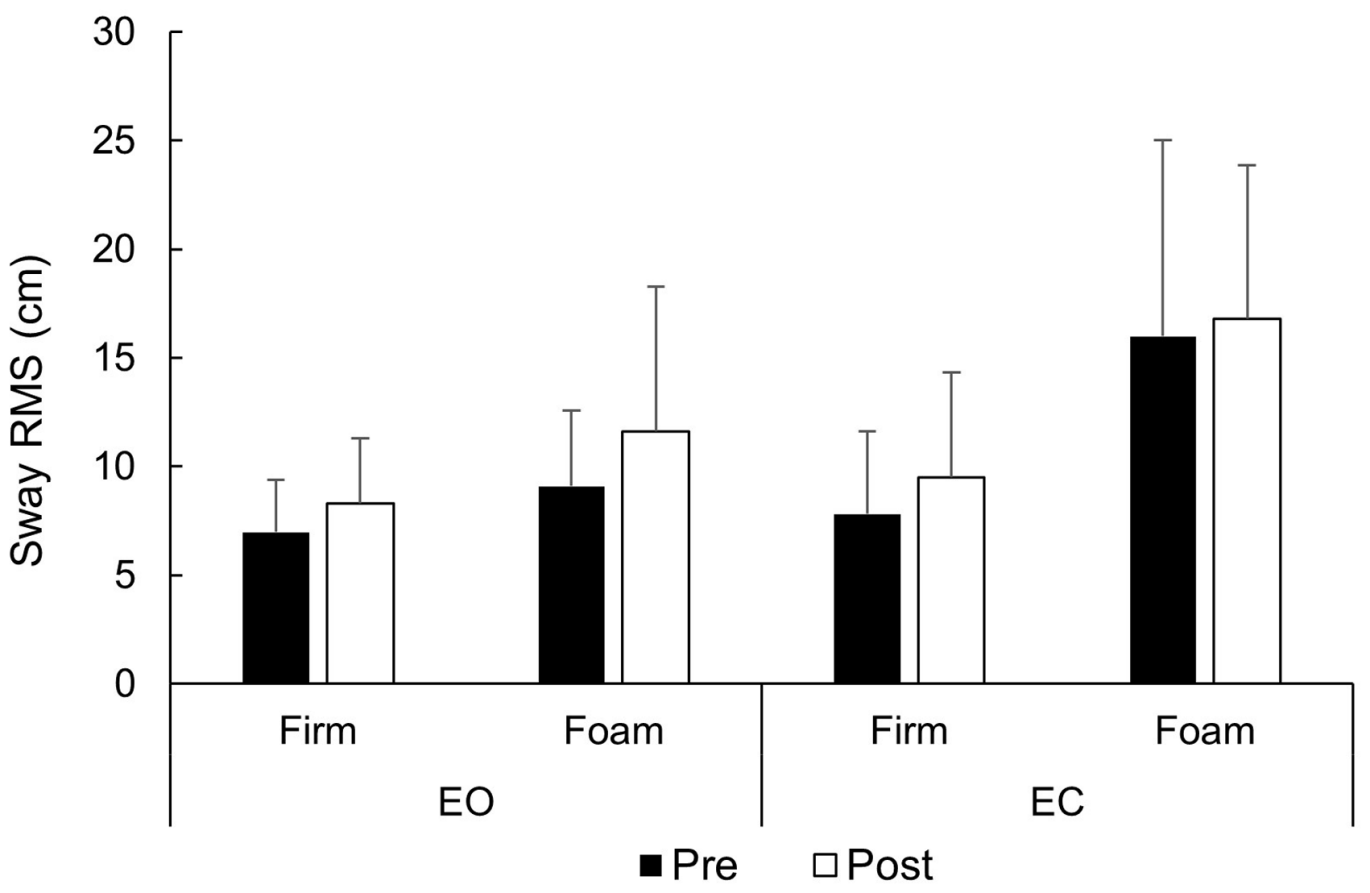
Sway velocity across vision, surface and fatigue conditions.

Regarding sway velocity (Figure 1), there was a statistically significant large effect of surface (*F* = 30.3; *p* < 0.001; ηp^2^ = 0.60), with lower velocity values on a firm surface (EMM = 14.0 cm/s; 95% CI = 11.6–16.5 cm/s) compared to the compliant surface (EMM = 22.9 cm/s; 95% CI = 15.6–24.7 cm). There was no statistically significant surface × fatigue (*F* = 0.07; *p* = 0.788) nor surface × vision (*F* = 3.12; *p* = 0.092) interactions, indicating that the surface effect was similar regardless of fatigue and vision condition. In addition, there were no statistically significant main effects of fatigue (*F* = 2.26; *p* = 0.148) and vision (*F* = 1.72; *p* = 0.207), as well as no statistically significant vision × time (*F* = 0.09; *p* = 0.759) and no surface × vision × fatigue (*F* = 0.18; *p* = 0.669) interactions. Overall, the results suggest that sway velocity is statistically significantly affected only by surface condition.

**Figure 2:**
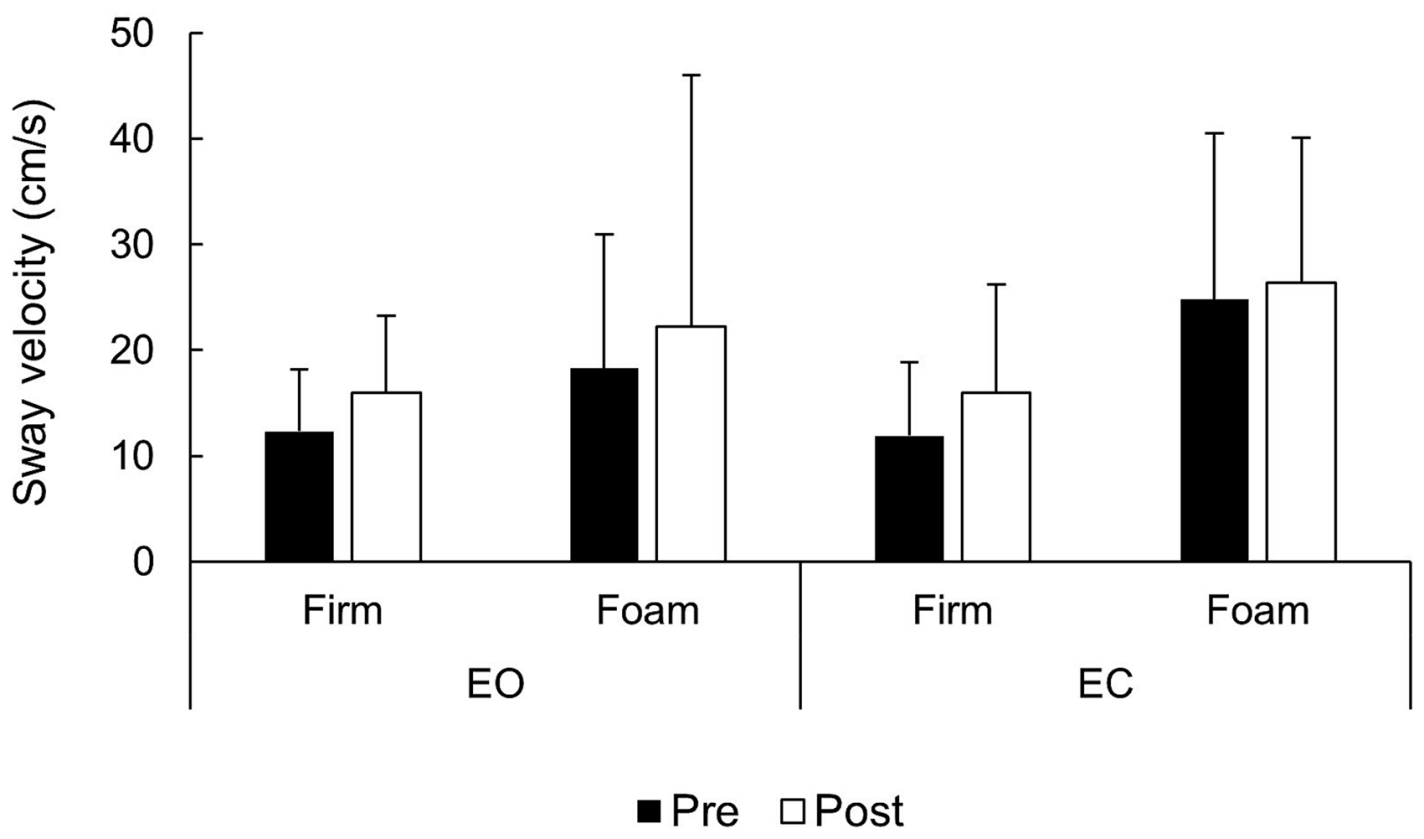
Sway RMS across vision, surface and fatigue conditions.

Regarding sway RMS (Figure 2), there were statistically significant and large effects of fatigue (*F* = 6.01; *p* < 0.023; ηp^2^ = 0.23), surface (*F* = 46.9; *p* < 0.001; ηp^2^ = 0.70), and vision (*F* = 16.7; *p* = 0.001; ηp^2^ = 0.46). Across surface and vision conditions, sway RMS increased from pre-fatigue (EMM = 10.0 cm; 95% CI = 8.2–1.7 cm) to post-fatigue (EMM = 11.6 cm; 95% CI = 9.7–13.4). Similarly, sway RMS was lower in the EO condition (EMM = 9.0 cm; 95% CI = 7.7–10.3 cm) compared to the EC condition (EMM = 12.5 cm; 95% CI = 10.1–14.9 cm). Finally, sway RMS was lower in the firm surface condition (EMM = 8.1 cm; 95% CI = 6.8–9.5 cm) compared to the compliant surface condition (EMM = 13.4 cm; 95% CI = 11.1–15.7 cm).

In addition to these main effects, there was a statistically significant and large vision × surface interaction (*F* = 23.7; *p* < 0.001; ηp^2^ = 0.54), necessitating further unifactorial analyses. In the EO condition, the surface effect was statistically significant and large (*F* = 12.3; *p* = 0.002; ηp^2^ = 0.38). Similarly, the effect of surface was statistically significant and large in the EC condition, with an even greater effect size (*F* = 53.8; *p* < 0.001; ηp^2^ = 0.73). There was no fatigue × vision (*F* = 0.38; *p* = 0.541), fatigue × surface (*F* = 0.014; *p* = 0.908) nor fatigue × vision × surface (*F* = 0.71; *p* = 0.407). Collectively, RMS results indicate an increase in EC, compliant surface, and post-fatigue conditions, compared to EO, stable surface, and pre-fatigue conditions, respectively. Surface and vision effect exhibited an interaction, which indicated that surface has a greater effect on sway RMS in the EC condition than in the EO condition. Finally, the fatigue effect on sway RMS was similar across vision and surface conditions.

## 4 Discussion

The aim of this study was to examine how fatigue, visual input, and surface stability interact to affect postural sway in patients with MS. Our main findings indicate that surface instability significantly increased both sway velocity and sway RMS, with participants demonstrating greater postural sway on a compliant surface compared to firm surfaces. Fatigue led to a significant increase in sway RMS but did not affect sway velocity. The absence of visual input (EC condition) also significantly increased sway RMS, and there was a significant interaction between vision and surface stability for sway RMS, showing that the effect of an unstable surface was more pronounced when visual input was absent. Fatigue increased postural sway independently of visual and proprioceptive manipulations, as no significant fatigue × sensory interactions were observed. This finding suggests that the destabilizing effect of fatigue was consistent across sensory conditions rather than being amplified by them. Despite balance deficits observed in objective measures, the relatively high ABC Scale scores suggest that participants did not perceive these balance issues as problematic in daily life, and low MFIS scores indicated that fatigue was not reported as a major concern. This suggests that, even in patients with mild disability (mean EDSS = 1.9), objective balance assessments can provide important insights into specific deficits that may not be detected in self-reported measures.

Our findings align with previous studies that have highlighted the challenges patients with MS face in maintaining postural stability under various conditions (Jallouli et al., 2022; McLoughlin et al., 2015; Van Emmerik et al., 2010). Our observed effect of fatigue on sway RMS (ηp^2^ = 0.23) represents a large effect and appears somewhat greater than the small-to-moderate effects (Cohen’s d ≈ 0.2–0.5) reported in earlier studies (see Sedaghati et al., 2023 for review). In contrast, the large effects of vision and surface in our study (ηp^2^ = 0.46 and 0.70, respectively) are in line with prior evidence that sensory manipulations dominate sway behavior compared to fatigue. While some previous research, such as that by Jallouli et al. (2022), reported that fatigue significantly increases postural sway, particularly when visual input is compromised (i.e., indicating a significant fatigue × vision interaction), we did not observe such an interaction in our study. Instead, we found that fatigue led to a significant increase in sway RMS regardless of the visual condition, suggesting that fatigue impairs postural control similarly whether or not visual input is available. Differences from the findings of Jallouli et al. (2022), who observed fatigue × vision interactions and gender-specific effects using a different fatiguing protocol, may be attributable to methodological and sample differences. Consistent with previous research (McLoughlin et al., 2015; Poh et al., 2017), an increase in postural sway was also found in the absence of vision. Previous studies also suggested that this increase is more pronounced in patients with MS compared to healthy controls, reflecting heightened reliance on vision (Fling et al., 2014). This is believed to compensate for deficits in the cerebellar system and associated proprioceptive function (Jackson and Bigelow, 2013; Peterka, 2002). Our study extends these findings by demonstrating that the absence of visual input and surface instability have an additive effect on sway RMS, emphasizing the compounded challenges these factors pose to postural control in patients with MS.

The underlying mechanisms of postural control deficits in MS likely involve disruption of sensory systems, impaired sensorimotor integration, and reduced motor abilities. Visual, vestibular, and proprioceptive impairments are all common in MS, with demyelination particularly affecting proprioceptive feedback and increasing reliance on vision for balance (Comber et al., 2018; Costello, 2016; Cochrane et al., 2021; Fling et al., 2014). Deficits in central integration of sensory inputs have also been reported (Jackson et al., 1995), and sensory integration training has been shown to improve balance outcomes (Gandolfi et al., 2015). Motor deficits, including reduced strength (Jørgensen et al., 2017) and impaired scaling of force production (Uygur et al., 2020; Uygur and Barone, 2023), further contribute to instability. Fatigue, which affects both neuromuscular and cognitive processes (Oliva Ramirez et al., 2021), may amplify these deficits by exacerbating muscle weakness, delaying postural reflexes, and overloading already compromised sensory integration (Moore et al., 2002).

The differential effects between sway RMS and sway velocity suggest that fatigue and absence of vision primarily increase the magnitude of postural deviations rather than the speed of corrective movements (Browne and O’Hare, 2001). One possible explanation is delayed activation of stabilizing muscles, leading to greater displacement but not necessarily faster corrections; however, this interpretation remains speculative as no neurophysiological measures were collected in the present study. Van Emmerik et al. (2010) also found no significant changes in sway velocity post-fatigue in patients with MS, supporting the notion that fatigue impacts the control of sway amplitude more than the dynamics of sway movements. In contrast, sway velocity was generally found to be more sensitive compared to sway amplitude in various test conditions (e.g., age, health status, and cognitive or proprioceptive challenges) in a previous study (Raymakers et al., 2005). This suggests that our findings may be MS-specific and reflective of disease-related changes in postural control mechanisms. From a clinical perspective, incorporating both sway RMS and sway velocity into balance assessments for MS could provide a more comprehensive understanding of postural instability, ensuring a more accurate evaluation of balance deficits.

## 5 Limitations

Some limitations of the study should be acknowledged. Our sample size was modest (n = 21), which reflects the strict inclusion criteria and recruitment feasibility within a single-center design (n = 21). This limits the generalizability of our findings to the broader population of patients with MS. A larger cohort would enhance the statistical power and allow for more robust conclusions. In particular, the relatively small sample size may have limited our ability to detect interaction effects (e.g., fatigue × vision). Second, the absence of a healthy control group prevents direct comparisons between patients with MS and individuals without neurological impairments. Including a control group in future studies would help determine whether the observed effects are specific to MS or also occur in the general population under similar conditions. Third, the fatigue protocol employed may not fully capture the complexity of fatigue experienced by patients with MS in daily life. MS-related fatigue is multifaceted, involving both physical and cognitive components (Oliva Ramirez et al., 2021). The 6MWT is a validated and reliable tool that induces motor fatigability in MS (Leone et al., 2016; Chen et al., 2021), making it appropriate for our focus on physical fatigue. However, it does not capture cognitive fatigue, which may also affect postural control. In addition, the fatigue protocol was not individually adjusted; while this ensured consistency across participants, it also resulted in variable fatigue levels, with some individuals reporting only low-to-moderate exertion. Future studies could incorporate more comprehensive fatigue-inducing tasks that mimic real-world activities to better understand fatigue’s impact on postural control. In addition, fatigue was assessed only through perceived exertion (RPE), and no objective physiological measures (e.g., heart rate) were collected. Future studies should include both subjective and objective indicators to provide a more comprehensive characterization of fatigue. Fourth, we focused on only two outcome measures: sway velocity and sway RMS. This decision was made due to our small sample size and the potential risk of Type I error. While these metrics are informative, they provide only a limited view of postural control. Incorporating additional measures, such as center of pressure trajectories, frequency-domain parameters, or nonlinear analyses, could offer a more detailed understanding of balance impairments in MS and should be considered in future studies.

## 6 Conclusion

This study indicates that fatigue exacerbates postural instability in people with MS, particularly when multiple sensory interactions are present. By jointly considering fatigue and sensory manipulations, we extend previous research that has mainly examined these factors in isolation. Clinically, these results highlight the importance of incorporating both fatigue protocols and complex sensory conditions into balance assessments, as such contexts better reflect the challenges that individuals with MS encounter in daily life. Measures such as sway RMS under unstable or no-vision conditions appear especially sensitive and should be prioritized when evaluating postural control in this population. These insights support the development of more ecologically valid assessment protocols that can improve the detection of balance impairments and better guide clinical decision-making.

## Data Availability

The datasets presented in this study can be found in online repositories. The names of the repository/repositories and accession number(s) can be found at: https://zenodo.org/records/15362811.
